# Goals of care conversation teaching in residency – a cross-sectional survey of postgraduate program directors

**DOI:** 10.1186/s12909-016-0839-2

**Published:** 2017-01-06

**Authors:** Amanda Roze des Ordons, Aliya Kassam, Jessica Simon

**Affiliations:** 1 Department of Critical Care Medicine and Division of Palliative Medicine, University of Calgary Cumming School of Medicine, South Health Campus ICU, 4448 Front St. SE, Calgary, AB T3M 1M4 Canada; 2 Office of Postgraduate Medical Education, Cumming School of Medicine, University of Calgary Cumming School of Medicine, Heritage Medical Research Building, Room G02 3330 Hospital Dr. NW, Calgary, AB T2N 4N1 Canada; 3 Division of Palliative Medicine, University of Calgary Cumming School of Medicine, Foothills Medical Center, South Tower, Room 710 1403 29th St. NW, Calgary, AB T2N 2T9 Canada

## Abstract

**Background:**

Residents are commonly involved in establishing goals of care for hospitalized patients. While education can improve the quality of these conversations, whether and how postgraduate training programs integrate such teaching into their curricula is not well established. The objective of this study was to characterize perceptions of current teaching and assessment of goals of care conversations, and program director interest in associated curricular integration.

**Methods:**

An electronic survey was sent to all postgraduate program directors at the University of Calgary. Quantitative data was analyzed using descriptive statistics and qualitative comments were analyzed using thematic analysis.

**Results:**

The survey response rate was 34% (22/64). Formal goals of care conversation teaching is incorporated into 63% of responding programs, and most commonly involves lectures. Informal teaching occurs in 86% of programs, involving discussion, direct observation and role modeling in the clinical setting. Seventy-three percent of programs assess goals of care conversation skills, mostly in the clinical setting through feedback. Program directors believe that over two-thirds of clinical faculty are prepared to teach goals of care conversations, and are interested in resources to teach and assess goals of care conversations. Themes that emerged include 1) general perceptions, 2) need for teaching, 3) ideas for teaching, and 4) assessment of goals of care conversations.

**Conclusions:**

The majority of residency training programs at the University of Calgary incorporate some goals of care conversation teaching and assessment into their curricula. Program directors are interested in resources to improve teaching and assessment of goals of care conversations.

**Electronic supplementary material:**

The online version of this article (doi:10.1186/s12909-016-0839-2) contains supplementary material, which is available to authorized users.

## Background

Residents are often involved in establishing goals of care with their patients, however, they may struggle with the complexity of the communication involved [1]. Goals of care conversations involve exploring and integrating a person’s illness experience, values and preferences with information about their medical condition to arrive at a decision that guides medical care [2]. The conversation should follow principles of informed consent, respect the patient or surrogate’s preference for decision-making, and include a medical recommendation integrating the current clinical situation with the patient’s values and wishes. The discussion and decision are translated into a plan of care and documented in the medical record [2]. Establishing goals of care has been cited as a core competency within both the CanMEDS and ACGME frameworks [3, 4]. Educational interventions involving seminars, group discussion, simulated practice and feedback have been shown to improve trainee skills and confidence in goals of care conversations [5–7].

When goals of care are either not discussed, or not addressed appropriately, the misunderstandings and intensity of care that exceeds that desired by the patient or is medically unwarranted has many consequences. For example, while resource overutilization is often cited, [8] perhaps of greater importance are the reduction in quality of life, the immediate and long-term psychological impact on patients and their families, [9] and the moral distress experienced by the healthcare team [10].

The provincial health service (Alberta Health Services) has a policy and procedure encouraging advance care planning and providing a medical order framework of “Goals of Care Designations” [11]. Despite this, many trainees at the University of Calgary, Alberta, Canada struggle with goals of care conversations. Recognizing the importance of these conversations and need for effective education, we were curious about factors contributing to trainee discomfort. As a first step in developing an institutional approach to integrating goals of conversation teaching into postgraduate medical education, we were interested in whether and how this topic was currently being addressed. The objective of this study was to describe goals of care conversation teaching and evaluation implemented by postgraduate training programs at the University of Calgary and to assess program directors’ interest in integrating new resources into existing curricula.

## Methods

An electronic survey based on a review of the literature was developed by the primary investigator (AR) and reviewed by two palliative care physicians, one of whom is also a physician consultant for advance care planning and goals of care designations (JS), contributing content validity; minor changes to wording were made as a result. The survey asked about formal and informal approaches to teaching and assessment of goals of care conversations, and program directors’ interest in future implementation of teaching and assessment of these conversations (Additional file 1). Formal teaching refers to strategies planned for in advance and delivered in the classroom setting; informal teaching refers to teaching that occurred in the clinical setting.

E-mail invitations to participate in the survey were sent to all postgraduate program directors (*n* = 64) at the University of Calgary from September 2014 to January 2015. The initial invitation and two reminders were sent to all program directors as a group; a final reminder was sent individually to program directors who had not yet responded.

Survey results were collated and quantitative data analyzed using Excel to compute descriptive statistics. Qualitative free-text responses were subjected to thematic analysis [12]. One of the investigators (AR) inductively developed a preliminary coding framework through multiple readings of free-text responses; the codes were applied to the data and organized into themes and subthemes.

## Results

### Quantitative

The survey response rate was 34% (22/64) overall, comprising 40% (6/15), 33% (12/36) and 23% (3/13) of adult surgical, adult medical, and pediatric medical/surgical programs, respectively. Formal and informal goals of care conversation teaching is incorporated into 63 and 86% of these programs, respectively. Formal curriculum time dedicated to goals of care teaching is 1–4 h/year and 4–8 h/year in 46 and 14% of these programs, respectively. Of formal teaching methods, didactic lectures are most common, role play and internet resources least common, and reflective writing not used. Informal teaching methods include discussion in the clinical setting, direct observation, and role modeling (Table 1). Goals of care conversation skills are assessed in 73% of programs; direct observation and feedback are the most common approaches to assessment. Written exams and multidisciplinary team assessment are least common (Table 1).

**Table 1:** Teaching and assessment of goals of care conversations

Teaching goals of care conversations	N (%)	Assessment of goals of care conversations	N (%)
Formal - didactic lectures	9/22 (41.0)	Direct observation and informal feedback	11/22 (50.0)
Formal - simulation	6/22 (27.3)	OSCE	4/22 (18.2)
Formal - small group discussion	5/22 (22.7)	Direct observation and feedback guided by form	4/22 (18.2)
Formal - role play	3/22 (13.6)	Case-based oral exam	2/22 (9.1)
Formal - internet resources	3/22 (13.6)	Written examination	1/22 (4.5)
Formal - reflective writing	0/22 (0)	Multidisciplinary team assessment	1/22 (4.5)
No formal GCC teaching	8/22 (36.4)	No assessment of GCC skills	6/22 (27.3)
Informal - discussion in clinical setting	18/22 (81.8)		
Informal - direct observation/feedback	16/22 (72.7)		
Informal - role modeling	14/22 (63.6)		
No informal GCC teaching	3/22 (13.6)		

*GCC* goals of care conversations, *OSCE* objective structured clinical examination

Sixty-eight percent of responding program directors believe clinical faculty are at least somewhat prepared to teach goals of care conversations; 13.6% believe clinical faculty are somewhat unprepared, and 18.2% believe faculty are not prepared for such teaching. Most program directors are interested in incorporating further goals of care conversation teaching (77%) and assessment (55%) into their programs.

### Qualitative

Four themes identified were general perceptions of goals of care conversations, need for goals of care conversation teaching, ideas for goals of care conversation teaching, and assessment of goals of care conversations. These themes and the corresponding subthemes and supporting quotes are provided in Table 2.

**Table 2:** Comments on teaching and learning about goals of care conversations

Theme	Subtheme	Quotes
General perceptions of GCC	A challenging conversation Residents responsible for majority of GCC GCC skills important for all residents Most residents already skilled in GCC Residents confident in GCC	“Can be a difficult topic in certain situations.” “Residents do the majority of goals of care discussions.” “Necessary learning for all residents in all programs!” “The majority [of residents] are reasonably good at this.” “I believe that our residents are comfortable discussing goals of care.”
Needs for GCC teaching	Gap in formal GCC teaching Uncertain quality of GCC teaching Formal GCC teaching would be valuable GCC teaching already well-integrated into curriculum	“It is in our objectives and is a gap in our education process.” “I suspect our current instruction around goals of care are somewhat variable in terms of quality.” “I think it would be valuable to add a more formal didactic component to how we teach this.” “I believe our program discusses goals of care and models this process very well through our preceptors.”
Ideas for GCC teaching	Tailored to discipline-specific needs Avoiding formulaic approaches Specific techniques	“Our needs might be a little different than other programs.” “Every situation is different… a ‘formulaic approach’ may be counterproductive.” Online modules, podcasts, practical tips, workshops, simulation, clinical exposure, role modeling, mentorship
Assessment of GCC	Current assessment methods artificial Standardized assessment tool would be valuable	“At times artificial measures of assessment of expertise in this area.” “A standardized tool for evaluating these discussions is an excellent idea.”

*GCC* goals of care conversations

## Discussion

Most postgraduate medical education programs at this institution incorporate some goals of care conversation teaching within their curricula; the majority of teaching and assessment takes place informally within the clinical setting. Previous research into needs for communication teaching has mainly focused on trainees; this project is unique in assessing postgraduate medical education program directors within an academic institution.

Many program directors identified formal goals of care conversation teaching as a gap in their curricula; others perceived that such teaching is already well-integrated into their respective programs. Programs that identified a gap were interested in methods to more consistently teach goals of care conversations, and proposed a number of creative ideas. For programs that already incorporate such teaching, evaluation of the quality and consistency of such teaching will be important.

Several program directors believed that residents are comfortable and highly skilled in discussing goals of care with patients and families, while research from other institutions has suggested otherwise. In the absence of formal goals of care conversation teaching, learning occurs through unsupervised practice and vicariously through observing senior trainees and faculty [13]. Furthermore, residents are infrequently observed or engaged in feedback conversations about their performance in these discussions [14–16]. Residents may also approach goals of care conversations in a scripted manner, with elements of the discussion and decision often misunderstood, and discrepancies between patients’ actual wishes and those documented [17, 18]. Residents perceive these conversations as difficult, often lack confidence in their own communication skills, and experience emotional distress [19, 20]. Assessment of residents’ perceptions and skills in having goals of care conversations will be important to verify or challenge program director perceptions within our local context.

Program directors also believed that most faculty are comfortable in teaching goals of care conversations. Faculty perceptions of their own competence in this area may be inaccurate, given the link between communication skills and self-concept, and limitations of self-assessment [21]. Resident assessment of faculty teaching and objective measures of the impact of teaching could motivate faculty to seek additional training in discussing goals of care and teaching these conversations.

It is interesting to note that programs at our institution do not use reflective writing to teach goals of care conversations. Reflection has been identified as critical in developing and maintaining competency in clinical reasoning as a medical expert, and in the communicator and, professional roles; [3] writing as a means of stimulating reflection has been shown to enhance self-reflection, personal and professional development, and empathy [22, 23]. Programs may find reflective writing a valuable method for teaching goals of care conversations.

Strengths of this study include the inclusion of both quantitative and qualitative data, allowing for both generalizations and elaboration of responses, respectively. Limitations include a low survey response rate and the single-centre focus of the study, such that the results are not generalizable outside of our local context. In addition, we did not elicit trainee and faculty perspectives; future research to obtain their perspectives will provide valuable information about the impact of current goals of care conversation teaching, gaps in current education, and how those gaps might be addressed..

## Conclusions

Teaching and assessment of goals of care conversations occurs most commonly in the clinical setting. Future study of residents’ perspectives and opinions of the many healthcare providers, patients and families they work with will broaden our understanding of the current educational milieu and allow us to tailor educational initiatives to trainee and program needs.

## Additional file

Additional file 1: Postgraduate residency program director survey. (DOCX 18 kb)
