# Supplementary material for: Goals of care conversation teaching in residency – a cross-sectional survey of postgraduate program directors
Source: BMC Med Educ. 2017 Jan 6;17:6. doi: 10.1186/s12909-016-0839-2 (PMC5217412; doi:10.1186/s12909-016-0839-2)
Supplement: Additional file 1: — Postgraduate residency program director survey. (DOCX 18 kb) [file 12909_2016_839_MOESM1_ESM.docx]

**Additional file 1: Postgraduate residency program director survey**

*A goals of care discussion is a complex communication process that occurs in an institutionalized setting*

*and that leads to a medical decision about life-sustaining therapies.*

We are seeking information about current goals of care discussion teaching across residency programs at the University of Calgary. We are interested in your responses to the following questions:

1. Residency program: __________________________________________________________________________________
2. Total number of residents in your program: _______________________________________________________
3. Is formal teaching about goals of care discussions incorporated into your residency training program?
   1. Yes (proceed to Question 4)
   2. No (proceed to Question 5)
4. Description of formal teaching and learning about goals of care discussions in your residency program:
   1. Formal methods of teaching and learning about care discussions currently incorporated into your residency training program include (select as many as applicable):
      1. Internet resources
      2. Didactic lectures
      3. Role play
      4. Simulation
      5. Other (please specify): ______________________________________________________________
   2. Number of hours per year dedicated to formal teaching about care discussions in your residency program:
      1. 1 – 3
      2. 4 – 6 hours
      3. 7 – 9 hours
      4. ≥ 10 hours
5. Is informal teaching about care discussions incorporated into your residency training program?
   1. Yes (proceed to Question 6)
   2. No (proceed to Question 7)
6. Informal methods of teaching and learning about care discussions currently incorporated into my residency training program include (select as many as applicable):
   1. Discussion on rounds or at the bedside
   2. Role modeling by faculty
   3. Direct observation of resident by faculty
   4. Other (please specify): ______________________________________________________________
7. Please indicate the current approach to assessment of resident competency in discussing goals of care with patients and their families (select as many as applicable):
   1. Written examination
   2. Objective Structured Clinical Examination (OSCE)
   3. Mini-Clinical Evaluation Exercise (mini-CEX)
   4. Supervision in clinical practice with feedback guided by a form
   5. Supervision in clinical practice with informal feedback
   6. Other (please specify): ­­­______________________________________________________________
   7. My program does not assess resident competency in Goals of Care discussions
8. To what degree to you think that faculty in your department are prepared to model and teach discussions about goals of care?

1 2 4 5

Not at all prepared Not prepared Prepared Very prepared

1. Would you be interested in incorporating a care discussions workshop into your resident program’s academic teaching?

1 2 4 5

Not at all interested Not interested Interested Very interested

- Please indicate reason for above response: ­­­­­­­­­

___________________________________________________________________________________________________________________________

___________________________________________________________________________________________________________________________

___________________________________________________________________________________________________________________________

1. Would faculty in your program value a tool to guide feedback when observing care discussions held by residents?

1 2 4 5

Not at all interested Not interested Interested Very interested

- Please indicate reason for above response: ­­­­­­­­­

___________________________________________________________________________________________________________________________

___________________________________________________________________________________________________________________________

___________________________________________________________________________________________________________________________

1. Is your program interested in being involved in the development of a tool to guide feedback when observing care discussions held by residents?

1 2 4 5

Not at all interested Not interested Interested Very interested

1. Please include other thoughts you may have about teaching and learning goals of care discussions :

___________________________________________________________________________________________________________________________

___________________________________________________________________________________________________________________________

___________________________________________________________________________________________________________________________

___________________________________________________________________________________________________________________________

___________________________________________________________________________________________________________________________

**Thank you for your participation in this survey; your opinions are highly valued.**
